# Community Health Workers Use Malaria Rapid Diagnostic Tests (RDTs) Safely and Accurately: Results of a Longitudinal Study in Zambia

**DOI:** 10.4269/ajtmh.2012.11-0800

**Published:** 2012-07-01

**Authors:** Helen Counihan, Steven A. Harvey, Masela Sekeseke-Chinyama, Busiku Hamainza, Rose Banda, Thindo Malambo, Freddie Masaninga, David Bell

**Affiliations:** Malaria Consortium, Maputo, Mozambique; Johns Hopkins Bloomberg School of Public Health, Baltimore, Maryland; Malaria Consortium, Lusaka, Zambia; Zambia National Malaria Control Center, Lusaka, Zambia; Livingstone District Health Management Team, Livingstone, Zambia; World Health Organization, Lusaka, Zambia; Foundation for Innovative New Diagnostics (FIND), Geneva, Switzerland

## Abstract

Malaria rapid diagnostic tests (RDTs) could radically improve febrile illness management in remote and low-resource populations. However, reliance upon community health workers (CHWs) remains controversial because of concerns about blood safety and appropriate use of artemisinin combination therapy. This study assessed CHW ability to use RDTs safely and accurately up to 12 months post-training. We trained 65 Zambian CHWs, and then provided RDTs, job-aids, and other necessary supplies for village use. Observers assessed CHW performance at 3, 6, and 12 months post-training. Critical steps performed correctly increased from 87.5% at 3 months to 100% subsequently. However, a few CHWs incorrectly read faint positive or invalid results as negative. Although most indicators improved or remained stable over time, interpretation of faint positives fell to 76.7% correct at 12 months. We conclude that appropriately trained and supervised CHWs can use RDTs safely and accurately in community practice for up to 12 months post-training.

## Introduction

Recent advances in point-of-care (POC) diagnostic technology offer tremendous potential for community-based infectious disease management. POC diagnostics could significantly increase the quality of basic health services for remote populations in low-resource settings. Currently, many such services are provided by community health workers (CHWs) with limited training and supervision.^1^,^2^ Realizing the potential of POC diagnostics at the community level will depend upon demonstrating that CHWs can prepare and interpret such tests accurately and safely.

POC diagnosis could be particularly useful for managing malaria. The cost of artemisinin-based combination therapy (ACT), growing anti-malarial resistance, and declining prevalence have all heightened recognition of the need for parasite-based diagnosis.^3^ The World Health Organization (WHO) now recommends parasite-based diagnosis for patients of all ages in all transmission zones.^4^ However, many suspected malaria cases occur far from microscopy-capable health facilities.^2^,^5^,^6^

Malaria rapid diagnostic tests (RDTs) make parasite-based diagnosis possible where microscopy is unavailable.^7^,^8^ RDTs are becoming a key component of community case management of malaria (CCMm), also known as home management of malaria (HMM).^9^,^10^ Access to accurate diagnosis and ACTs close to home should reduce malaria-related deaths, especially among young children. Parasite-based malaria diagnosis at the community level could also serve as the first step toward better management of non-malarial causes of febrile illness, some equally life-threatening.^11^,^12^

However, parasite-based diagnosis at community level presents challenges, particularly in Africa where almost 90% of the world's malaria fatalities occur.^3^,^13^ RDTs require that health workers obtain a finger-prick blood sample, follow a simple but strict preparation procedure, and accurately interpret results. Concerns about blood-borne disease and CHW competence have made many African health systems reluctant to permit RDT use by CHWs. Previous studies show CHWs can use RDTs accurately and safely after a brief training period with support from a well-designed set of pictorial instructions (a “job-aid”).^14^,^15^ However, these studies assessed CHW performance in a health facility immediately after training. Until now, there has been little evidence about CHW capability to maintain adequate performance over time at the village level.

This work reports results from a 12-month longitudinal study of RDT use by Zambian CHWs. The objective was to track participant performance over time, assess whether safety and effectiveness remained adequate up to 1 year post-training, and thus determine the advisability of incorporating RDTs as part of HMM.

## Methods

### Study design.

The study ran from November 2007 to December 2008. To assess performance over time, the study team recruited 66 CHWs from Livingstone District, Southern Province, Zambia. Each CHW received a job-aid and a half-day training on RDT use and interpretation based upon a previously validated curriculum.^14^,^16^ The CHWs who successfully completed the training returned home with a copy of the job-aid, an estimated 3-month supply of RDTs (ICT Malaria Pf Cassette Test (ML01); ICT Diagnostics, Cape Town, South Africa) and ACT (Coartem) plus gloves, cotton wool, a sharps disposal box, and a plastic bucket with lid for non-sharps disposal. All participating CHWs received instructions on what to do in case of a finger prick or blood contamination accident, including where to obtain human immunodeficiency virus (HIV) prophylaxis. The district level Ministry of Health provided CHWs with paracetamol for treating fevers in RDT-negative patients. The CHWs received home visits within 2 weeks of the training to ensure they were following safety precautions and not endangering themselves, their patients, or other community members. Data collected during this initial visit were not included in the final study analysis. Thereafter, a trained observer visited each CHW at 3, 6, and 12 months post-training. Observers used a standardized checklist to assess each CHW's performance in preparing the test. Whenever possible, CHWs were observed testing an actual febrile patient. However, because of the distances between some villages and the need to complete each wave of observation in a timely manner, there were some cases in which no febrile patient was available for testing when the observer arrived. In such cases, observers assessed CHW performance using a non-febrile volunteer. Observers also used a photograph of 10 RDTs with positive, negative, and invalid results to assess CHW ability to read RDTs accurately. To avoid learning effect, the order of the photographs was varied at each observation.

The study was conducted in collaboration with the Zambia National Malaria Control Center (NMCC) and as part of Zambia's first HMM pilot. Participating CHWs worked within the catchment area of eight rural health facilities. Because the study was designed to assess CHW performance in as close as possible to real-life conditions, nearby health clinics assumed responsibility for resupply. The clinic and district health management team (DHMT) staff was encouraged to continue routine supervision. At the 3-month follow-up, each CHW received a poster-sized version of the job aid and a photographic guide explaining how to interpret RDT results.

### Observation.

Observers received 2 days of training in observation techniques including instruction on how to minimize observer-induced reactivity bias. Throughout most of the 3- and 6-month observations, study co-investigators accompanied the observers. The checklist divided test preparation into 19 steps. Observers noted whether the CHW performed each step correctly, incorrectly, or not at all. Each observer also noted whether he or she missed observing a step. Steps were divided into two categories: those critical to safe and accurate performance and those that would not jeopardize patient, CHW, or community safety, if performed incorrectly or missed. Table 1 lists each step with critical steps in bold type. We defined safe use to consist of correct completion of steps 9 and 11 (use a sterile lancet and dispose of the lancet in an approved sharps container immediately after use). We defined accurate use as correct completion of steps 12, 13, 15, 16, and 17; all those necessary to arrive at a correct diagnosis. Four situations were deemed sufficiently serious to require immediate observer intervention and notification of study supervisors: attempting to use a lancet or pipette on more than one person, failure to adequately dispose of a lancet, incorrectly reading an actual test result, and failure to treat a positive result. The CHW was then asked to read the results of 10 RDT photographs presented on a card. At each observation, the observer recorded number of RDTs performed since the previous visit, number of positives, and number of patients < 5 years of age. Finally, the observer asked CHWs about any challenges or concerns they were experiencing.

### Data analysis.

Data were double entered into a Microsoft Access database (Microsoft, Redman, WA), checked for discordance, and then analyzed using Stata (College Station, TX). Percentages of total steps, crucial steps, and non-crucial steps performed correctly were calculated for each observation cycle. Generalized estimating equations (GEE) logistic regression models for panel data were fit to identify factors associated with correct performance of at least 90% of total test steps.^17^ The GEE models were also fit to identify factors associated with correct performance of 100% of critical steps and at least 90% of non-critical steps. For the reading of the 10 photographic results, performance was assessed for each observation period by calculating the pooled mean and median percentage of total tests read correctly and the percentage of CHWs who read each individual test correctly. Correctly interpreting photos of invalid RDT results was a particular concern: some readers incorrectly interpret the appearance of a test line but no control line as negative. For this reason, Stata's *pretest* command was used to estimate equality of proportions between invalid results showing no line versus invalid results showing only a test and no control line.^18^ Similarly, some users misread RDTs with a faint positive line as negative. The *t* test was used to determine whether there was a significant difference between correct interpretation of strong and faint positive test lines.

### RDT quality assurance.

Before the study, the RDT batches were lot tested according to WHO protocol at the Research Institute for Tropical Medicine (RITM) in the Philippines.^19^ At the end of the study, during the last round of observations, two RDTs were collected from each of 42 CHWs (all those who had sufficient RDTs in stock) for further quality control testing at RITM. Results showed 100% detection at 200 parasites/μL, indicating no measurable sensitivity loss despite prolonged storage in homes of participating CHWs.

### Ethical approval.

This study received ethical approval from WHO/TDR (RPC 167) and the Tropical Disease Research Center Ethics Committee, Ndola, Zambia. By the start of Phase III, Zambia had decided to include RDTs for confirmed diagnosis of malaria as part of HMM. Thus community-based RDT use became a routine rather than an experimental practice.

## Results

### Participant demographics.

Sixty-six CHWs participated in the training in November 2007 using the previously developed manual. One CHW was dropped from the study after failing to demonstrate sufficient competency during practice sessions. Thus, 65 were initially enrolled in the 12-month surveillance. At the 3-month observation period, two of these 65 were unavailable because of work or travel. At the 6-month observation one of these two was available, but the second had dropped out completely and three others were unavailable, giving a total of 61 CHWs observed. At 12 months, the CHW who returned at the 6-month observation was missing again, and four others were unavailable due to illness or bereavement, therefore the final observation included 59 CHWs. Table 2 presents participant socio-demographic characteristics.

### Test preparation.

**Figure 1.**
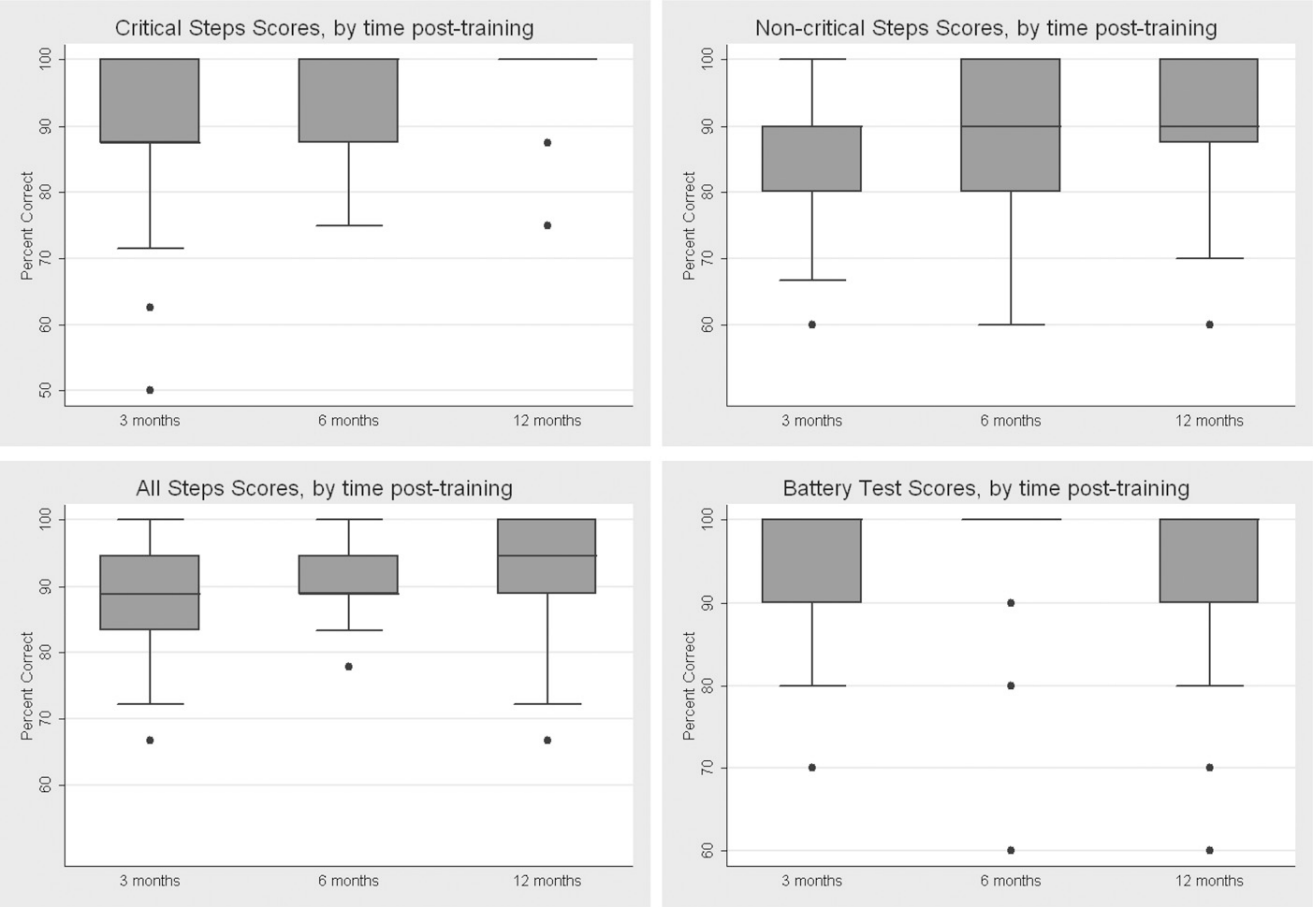
Percent of rapid diagnostic tests (RDT) steps completed or interpreted correctly at 3, 6, and 12 months post-training.

As shown in Figure 1, median CHW performance remained steady or improved over time for critical steps, non-critical steps, and RDT interpretation. The median percentage of critical RDT steps performed correctly rose from 87.5% at 3 months to 100% at 6 and 12 months. From the perspective of changes in individual score, performance on critical steps improved over time: 40.3% of CHWs (25 of 62) performed all critical steps correctly at 3 months compared with 61.7% (37 of 60) at 6 months, and 79.7% (47 of 59) at 12 months. If we broaden this group to include CHWs who made only one error, it includes 79% of participants (49 of 62) at 3 months compared with 90% (54 of 60) at 6 months and 97% (57 of 59) at 12 months. Table 3 provides step-by-step performance of both critical and non-critical steps at 3, 6, and 12 months.

On the basis of GEE logistic regression, CHW age and time since training (3, 6, or 12 months) were the only factors significantly associated with correct performance of all critical steps (Table 4). At 6 months post-training, CHWs were 2.4 times more likely to perform all critical steps correctly compared with the 3-month assessment (*P* = 0.02). At 12 months, they were 6.4 times more likely to perform all critical steps correctly compared with the 3-month assessment (*P* < 0.001). Compared with those 40 and under, CHWs 50 years of age and older were 62% *less* likely to score 100% on critical steps (*P* = 0.04).

### Safety.

In general, CHWs applied appropriate blood safety measures. All CHWs wore gloves, although at 3- and 6-month observations, two used a single pair of gloves on more than one patient, in one case due to insufficient supply. Lancet use was generally satisfactory. Once during the 3-month observation, an observer intervened when one CHW appeared to be about to re-use a lancet on a second patient. In all other cases, the only errors were setting the unused lancet down on its wrapper before use or setting the used lancet down on the table before disposing of it in the sharps box. At the 6-month observation, one CHW disposed of her lancet with her regular waste because she had no sharps box. The CHWs were instructed during training to report needle-stick injuries immediately to receive post-exposure prophylaxis for HIV, but no such injuries were observed or reported during the 12-month period.

### Errors in RDT use.

Median scores on most study measures improved over the surveillance period. One exception, as measured by the different test result photographs, was the ability to correctly interpret faint positive test lines as positive. This improved from 89.7% at 3 months to 96.7% at 6 months, and then declined to 76.7% at 12 months.

Slightly less than half of the participants collected the correct amount of blood during the 3-month observation; this rose to slightly over 60% during the 6- and 12-month observations. In most cases, participants collected too little blood rather than too much. As in other studies, CHWs found the blood collection device included with the RDT—in this case a disposable plastic pipette—difficult to use. Early on, CHWs also had difficulty transferring the blood to the test cassette: at the 3-month observation, eight of the 64 participants (13%) transferred most of the blood to the well wall rather than the absorbent pad. However, this error occurred in only three out of 60 of cases (5%) at the 6-month observation. At 12 months all participants transferred the blood correctly. Finally, three CHWs read test results incorrectly at 3 months, compared with only one at 6 and 12 months—with all but one being cases of reading faint positives as negative.

### Job-aid use.

Documenting job-aid use by CHWs was difficult. In many observations, the job-aid was present (e.g., hanging on a wall or tree), but the observer could not reliably determine whether the CHW referred to it while preparing or interpreting the test. Many CHWs said they avoided using the job-aid because they believed the observer wanted to test their ability to use the RDT without support. The notable exceptions were CHWs who used the job-aid to explain test procedures and possible results to patients before beginning the test.

### Accuracy at interpreting RDT results.

Using the photograph of 10 different RDT results, CHWs correctly identified 96.5% of positive tests at 3 months and 98.3% at 6 months, but only 90.5% at 12 months. Similarly, CHWs correctly identified 94.3% of negative results at 3 months, 97.9% at 6 months, and 94.7% at 12 months. Interpretation of invalid test results improved from 90.2% correct at 3 months to 96.7% at 6 months and 96.5% at 12 months.

Each photograph of 10 tests included two invalid results, one illustrated by no line (neither test nor control) and one illustrated by a test line only (no control line). As shown in Table 5, a significantly higher proportion of participants at 3 months correctly interpreted the RDT with no line than the RDT with a test line only. This difference remained significant, though smaller, at 6 months, but was no longer significant at 12 months. The CHWs consistently read photographs of strong positive test results correctly. Faint positives were more problematic, most notably at the 12-month observation.

### Supplies and supervision.

Many participating CHWs had difficulty obtaining additional RDTs and supplies from associated health facilities after exhausting initial stocks. In some cases, this occurred because the health facilities themselves experienced stock-outs. In other cases, facility managers may have been resistant to CHW use of RDTs as they were not informed by their supervisor who attended the introductory workshop. Occasionally, there were discrepancies in reporting of stock sufficiency between supervisors and CHWs within their area. Consequently, some CHWs experienced stock-outs of RDTs, drugs, and supplies until the study team resupplied them. To the best of our knowledge, no supervisory visits by health facility or DHMT staff occurred during the study period.

## Discussion

The study demonstrated consistently high performance over 12 months by CHWs using malaria RDTs after receiving a half-day competency-based training and a field-tested job-aid. The study also revealed a few instances of poor practice, with some participants repeating the same or similar errors throughout the 12-month period. In particular, difficulty reading faint positive test lines could lead to false-negative results. Previous studies have noted both this difficulty and the difficulty manipulating blood transfer devices.^14^,^20^–^23^

The study did not attempt to assess the frequency with which supervision visits or retraining should occur, but a supervisor should visit to address any high-risk behaviors soon after training a new cadre of CHWs. Regular visits thereafter seem warranted, particularly for CHWs demonstrating inadequate performance. Resupply provides an opportunity for skill reinforcement—in the CHW's normal environment if a supervisor brings supplies to the village, or at a health facility if the CHW visits regularly to collect supplies and submit data. Other studies on RDT use have also noted the need for ongoing training and supervision.^23^

The drop in correct interpretation of faint test lines at 12 months suggests that supervisory visits should stress this, and should occur at least semi-annually. Training programs should reinforce the distinction between test and control lines, ensure that CHWs make that distinction accurately, and emphasize that even faint lines indicate a positive result.

CHWs using the same pair of gloves on more than one patient were providing direct support to a health facility where this was considered normal practice if the gloves had no blood on them. More extensive training or supervision of health facility staff, and better supply of such commodities, appear to be needed in that case.

Improvement in the design of blood collection devices might address some of the problems of RDT use encountered in this study, in particular the reliability of volume transferred.^20^–^22^ In addition, initial training and supervision should emphasize the importance of collecting the correct volume of blood. This should include ensuring that trainees practice with whatever blood collection device will be packaged with their RDT(s) until they are able to collect the required volume of blood correctly every time. Retractable lancets would also reduce the significance of the (rare) lancet mishandling observed.

Although CHWs may prepare and interpret RDTs safely and accurately, acting on the result is fundamental to good outcomes. This study did not specifically assess CHW adherence to RDT results, but a retrospective assessment of drug consumption, to be published elsewhere, indicates strong compliance with prescription of ACTs.^24^ During follow-up visits, prescription of anti-malarials to RDT negative cases was observed by two CHWs at 3 months, but was no longer an issue during later visits. It is worth noting that the prevalence of malaria remained low throughout the study period, which resulted in some CHWs expressing concern that the RDTs may not work correctly. Despite this, they continued to use RDTs and only treat positive cases, as trained.

The observed errors led to minor modifications to the training manual, with increased emphasis on aspects of blood safety and RDT reading. Generic versions of the resulting materials are available on the WHO malaria RDT website.^16^ A library of product-specific versions is available from FIND.^23^

### Limitations.

Some limitations may have influenced study results. First, Livingstone district is economically better off than many areas of Zambia. Livingstone was chosen as a study site because it was the first district to implement HMM. Consistent with Livingstone's relatively higher socioeconomic status, 75% of participating CHWs had completed some secondary education. This could have produced better results than might occur with less educated CHWs.

The repeated visits by observers may also have influenced results. Initially, some CHWs were uncomfortable with preparing the RDT in front of an observer, which may have affected the quality of their performance. Over time CHWs became more comfortable with the visit procedure. To measure performance as accurately as possible, observers were instructed not to interfere with or correct a CHW unless that CHW was about to do something that presented an immediate danger to him- or herself, the patient, or the community. At times the observers tended to err on the side of intervention in ambiguous situations, avoiding harm to participants rather than preserving data integrity. Thus, although CHWs did not receive any retraining during the 12-month surveillance, they did receive some correction from observers from time to time.

Use of volunteers rather than febrile patients for some observations could have biased results. This was sometimes necessary because each observation cycle had to be completed within a short period, and febrile patients were not always available. However, the testing procedure is the same whether performed on a patient or a volunteer. Furthermore, because many CHWs lived in remote areas, the study team notified them one day before each observer visit to ensure they would be home for the observation. Having observers arrive unannounced would have been preferable from a methodological standpoint, but would have greatly reduced the probability of finding the CHW at home and available.

## Conclusions

With a well-designed job-aid and half-day training, CHWs can diagnose malaria safely and accurately using RDTs. CHWs participating in the study retained these skills in community practice up to 12 months post-training. However, occasional errors occur, therefore supervisors should conduct periodic performance appraisals and make necessary corrections to ensure patient, CHW, and community safety. The results offer good evidence for the appropriateness of CHWs taking finger-prick blood samples and using POC diagnostic tests in the community, provided they receive adequate training, job-aids, supplies, and follow-up supervision.

## Figures and Tables

**Table 1:** The 19 steps required to correctly and safely prepare a rapid diagnostic test (RDT) (critical steps noted in boldface type)

1. Assemble new test packet, swab, buffer, pipette, lancet and gloves.
**2. Put on new pair of gloves.**
3. Check expiry date on package.
4. Check desiccant sachet is still dry (do not include answer in total score).
5. Write patient's name on cassette.
6. Place cassette on a level surface.
**7. Clean finger with antiseptic/alcohol.**
8. Allow finger to dry before pricking it.
**9. Use a sterile lancet for finger prick.**
10. Puncture the side of the ball of the finger.
**11. Dispose of lancet in sharps bin immediately after pricking finger.**
12. Collect blood with the enclosed pipette making sure to fill close to the first cross line.
**13. Using the pipette, blot blood onto the pad in the smaller well.**
14. Dispose of pipette in sharps container immediately.
**15. Dispense 5 drops of clearing buffer into the larger well.**
16. Wait 15 minutes before reading *negative* results.*
**17. Read test results correctly.**
18. Record results in CHW register.
19. Dispose of non-sharps (gloves, wrappers, alcohol swab, and desiccant) safely.

* Positive results may be read before 15 minutes if control line has also appeared. Results should not be read after 30 minutes.

**Table 2:** Socio-demographic characteristics of participating CHWs

Characteristic	n (% or range)
Sex		
Male	27 (42.9)
Female	36 (57.1)
Mean age (years)	43.9 (18–64)
Education	
Some primary	4 (6.4)
Complete primary	6 (9.5)
Some secondary	47 (74.6)
Complete secondary	6 (9.5)
Prior malaria treatment experience	51 (81.0%)
Median months malaria treatment experience*	3 (0–348)*
Prior malaria RDT experience	2 (3.1%)

* A few community health workers (CHWs) with extensive malaria treatment experience result in a mean (31.3 months) that is not representative of the group as a whole. Thus, median is presented here instead of mean.

**Table 3:** Step-by-step performance: percent of CHWs who performed each step correctly at each observation

	3 months (*n* = 63)	6 months (*n* = 61)	12 months (*n* = 59)	Most common reasons for errors
Critical Steps				
Wearing gloves	90.3	98.3	100	Did not change gloves between patients
Clean finger before pricking	98.4	100	98.3	Skipped
Use sterile lancet	87.1	95.0	100	Tip of lancet touching gloves or work surface before pricking
Dispose of lancet safely	96.8	96.7	98.3	Set used lancet on table before disposing of it in sharps box
Blot blood correctly	87.1	95.0	100	Most blood went on wall of well
Dispense buffer drops accurately	74.2	83.3	89.8	Too many or too few drops
Wait correct amount of time	82.3	85.0	91.4	
Read RDT results correctly	95.1	98.3	98.3	Faint positive called negative
Non-critical steps				
Assemble everything before starting	93.6	78.3	94.9	
Check expiry date	96.8	100	96.6	
Write patient's name on cassette	90.3	95.0	96.6	
Place RDT on a level surface	100	100	98.3	
Allow finger to dry before pricking	98.4	91.7	98.3	
Prick side of finger	74.2	73.3	78.0	Pricked middle of finger instead
Collect blood correctly	53.2	63.3	61.0	Too little blood in most cases
Dispose of pipette correctly	98.4	96.7	98.3	
Record result in register	77.4	86.7	91.1	Usually because the subject was not a real patient
Dispose of non-sharps correctly	93.6	98.3	96.5	

* CHWs = community health workers; RDT = rapid diagnostic test.

**Table 4:** Adjusted odds ratios for a score of 100% on critical steps (GEE logistic regression model)*

	Adjusted odds ratio (95% CI)	*P* value
Time post-training		
3 months	1.00 (referent)	
6 months	2.39 (1.13–5.09)	0.02
12 months	6.42 (2.73–15.10)	< 0.001
CHW Age		
40 years and under	1.00 (referent)	–
41–49 years	0.61 (0.24–1.56)	0.30
50 years and above	0.38 (0.15–0.98)	0.04

* GEE = generalized estimating equations; CI = confidence interval.

**Table 5:** Correct interpretation of rapid diagnostic test (RDT) results (percent read correctly in each category by time post-training)

Ambiguous tests: no line vs. test line only					Positive test results: strong vs. faint positive line			
Time post-training	No line	Test line only	Difference	*P* value*	Strong positive	Faint positive	Difference	*P* value*
3 months	96.8	83.9	12.9	0.02	100	89.7	10.3	0.001
6 months	100	91.8	8.2	0.02	100	96.7	3.3	0.10
12 months	98.3	94.9	3.4	0.31	100	76.7	23.3	< 0.001

* *t* test for equality of proportions.

Over the 12-month surveillance period, there were four consistently poor performers. Two of these incorrectly interpreted 4 tests out of 30 total (13.3%) incorrectly, two incorrectly interpreted 5 out of 30 (16.7%) incorrectly, and one incorrectly interpreted 7 (23.3%) incorrectly.
